# Supplementary material for: Complex‐centric proteome profiling by SEC‐SWATH‐MS
Source: Mol Syst Biol. 2019 Jan 14;15(1):e8438. doi: 10.15252/msb.20188438 (PMC6346213; doi:10.15252/msb.20188438)
Supplement: Supplementary file 7 — Dataset EV6 [file MSB-15-e8438-s007.zip › feature_plots_bioplex/Q96DI7.pdf]

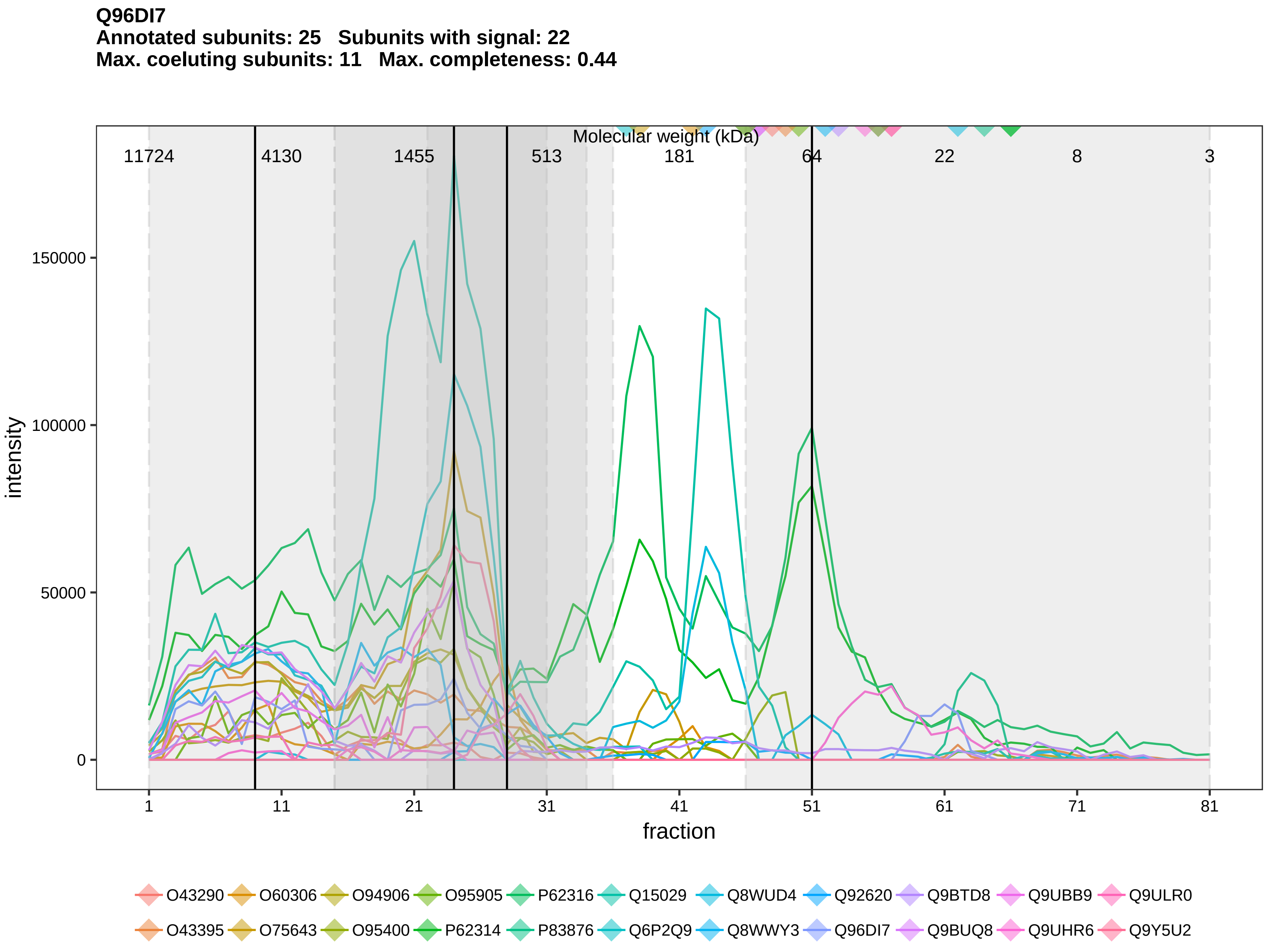
Q96DI7
Annotated subunits: 25 Subunits with signal: 22
Max. coeluting subunits: 11 Max. completeness: 0.44
Molecular weight (kDa)
11724
4130
1455
513
181
64
22
8
3
intensity
150000
100000
50000
0
1
11
21
31
41
51
61
71
81
fraction
O43290
O60306
O94906
O95905
P62316
Q15029
Q8WUD4
Q92620
Q9BTD8
Q9UBB9
Q9ULR0
O43395
O75643
O95400
P62314
P83876
Q6P2Q9
Q8WWY3
Q96DI7
Q9BUQ8
Q9UHR6
Q9Y5U2
